# Multidimensional Scaling of Cognitive Ability and Academic Achievement Scores

**DOI:** 10.3390/jintelligence10040117

**Published:** 2022-12-01

**Authors:** Em M. Meyer, Matthew R. Reynolds

**Affiliations:** 1 Department of Counseling, School Psychology and Family Science, College of Education, University of Nebraska, Kearney, NE 68849, USA; 2 Department of Educational Psychology, School of Education and Human Sciences, University of Kansas, Lawrence, KS 66045, USA

**Keywords:** multidimensional scaling, intelligence, academic achievement, complexity

## Abstract

**Author Note:**

Standardization data and analysis from the Kaufman Test of Educational Achievement, Second Edition-Comprehensive Form (KTEA-2). Copyright © 2004 NCS Pearson, Inc. Used with permission. All rights reserved. Standardization data and analysis from the Kaufman Assessment Battery for Children, Second Edition (KABC-II). Copyright © 2004 NCS Pearson, Inc. Used with permission. All rights reserved. Standardization data from the Wechsler Intelligence Scale for Children, Fifth Edition (WISC-5). Copyright © 2014 NCS Pearson, Inc. Used with permission. All rights reserved.

**Abstract:**

Multidimensional scaling (MDS) was used as an alternate multivariate procedure for investigating intelligence and academic achievement test score correlations. Correlation coefficients among Wechsler Intelligence Scale for Children, Fifth Edition (WISC-5) and Wechsler Individual Achievement Test, Third Edition (WIAT-III) validity sample scores and among Kaufman Assessment Battery for Children, Second Edition (KABC-II) and Kaufman Test of Educational Achievement, Second Edition (KTEA-2) co-norming sample scores were analyzed using multidimensional scaling (MDS). Three-dimensional MDS configurations were the best fit for interpretation in both datasets. Subtests were more clearly organized by CHC ability and academic domain instead of complexity. Auditory-linguistic, figural-visual, reading-writing, and quantitative-numeric regions were visible in all models. Results were mostly similar across different grade levels. Additional analysis with WISC-V and WIAT-III tests showed that content (verbal, numeric, figural) and response process facets (verbal, manual, paper-pencil) were also useful in explaining test locations. Two implications from this study are that caution may be needed when interpreting fluency scores across academic areas, and MDS provides more empirically based validity evidence regarding content and response mode processes.

## 1. Introduction

Individually administered, norm-referenced intelligence and academic achievement tests are important components of comprehensive psychoeducational evaluations (Benson et al. 2019; Rabin et al. 2016). Scores from these tests provide evidence of expected learning (intelligence), demonstrated learning (academic achievement), and cognitive and academic strengths and weaknesses (Benson et al. 2019). Results from these tests are combined with other assessment information to make diagnoses, assist with educational eligibility decisions, and plan individualized educational programs and interventions (Lichtenberger and Breaux 2010; Mather and Abu-Hamour 2013). Psychologists depend upon up-to-date information to improve the likelihood of valid interpretations of their scores.

### 1.1. Intelligence and Academic Achievement Tests Are Multidimensional and Related

Individually administered intelligence tests measure general intelligence and more specific abilities such as problem-solving, auditory processing, and memory (Kaufman and Kaufman 2004; Wechsler 2014). The latent structure of intelligence is hierarchical and multidimensional (Caemmerer et al. 2020; Reynolds and Keith 2017), and it is organized as such in Cattell-Horn-Carroll (CHC) theory (McGrew 2009; Schneider and McGrew 2018). CHC abilities such as novel problem solving, auditory processing, and memory give rise to observable differences in intelligence test scores. These CHC abilities are interrelated and correlate with a general factor of intelligence, or *g*. Fluid reasoning (Gf) typically has the strongest correlation with *g* (Carroll 1993; Jensen 1998). Other broad abilities include comprehension-knowledge (Gc), short-term working memory (Gsm or Gwm), long-term storage and retrieval (Glr), visual processing (Gv), and auditory processing (Ga).

Individually administered academic achievement tests measure acquired skills in academic domains such as reading, writing, and math. Within each domain, there are levels of complexity: basic skills (e.g., knowledge of letter-sound relations), fluency (e.g., automatic word reading), and higher-order thinking (e.g., reading comprehension). Composite achievement test scores can reflect performance within a domain or across domains according to complexity (Mather and Wendling 2015). Acquired knowledge, including the types of skill domains measured with academic achievement tests such as reading and writing or math, are also described in CHC theory (Schneider and McGrew 2018). 

A valid interpretation of intelligence and academic achievement scores depends on understanding how and why the test scores correlate with each other. Most studies of intelligence and academic achievement test score correlational structures have used structural equation modeling, factor analysis, and regression analysis. Correlations between *g* and latent general academic achievement are very strong (Kaufman et al. 2012). Intelligence, however, contributes to specific academic achievement domains generally and via specific CHC abilities (Floyd et al. 2007; Gustafsson and Balke 1993; Niileksela and Reynolds 2014). For example, verbal comprehension (understanding words and their relations) and auditory processing (perception and manipulation of sound) affect reading in addition to the effects of *g* (Garcia and Stafford 2000; Keith 1999; Vanderwood et al. 2002). 

CHC broad abilities influence both specific and broad areas of reading, math, and writing (Benson et al. 2016; Caemmerer et al. 2018; Cormier et al. 2016; Cormier et al. 2017; Floyd et al. 2003; Hajovsky et al. 2020; McGrew and Wendling 2010; Niileksela et al. 2016). As such, tests that draw upon CHC abilities relate to tests of specific academic achievement. In addition, intelligence and academic achievement tests may share characteristics that contribute to their correlations such as task complexity (basic skills, fluency, or higher-order thinking or problem-solving), stimuli (words, numbers, or pictures), and examinee response modes (oral response, manipulation of materials, or written). Empirical studies of the correlations between intelligence and achievement scores should try to incorporate these other shared characteristics. 

### 1.2. Validity Evidence from Multidimensional Scaling

AERA, APA, and NCME Standards for Educational and Psychological Testing (American Educational Research Association et al. 2014) include test content and response processes as sources of validity evidence. These types of evidence, however, are rarely demonstrated with data, rather they are described by conducting an alignment study or expert panel review. Multidimensional scaling (MDS) is an unrestricted, multivariate technique for analyzing correlations and exploring test score interrelations in a visual way. It is also an empirical method used to evaluate validity evidence based on content (Li and Sireci 2013) and response process (Cohen et al. 2006).

MDS has been applied to intelligence test scores alone (Cohen et al. 2006; Guttman and Levy 1991; McGill 2020; Meyer and Reynolds 2018; Tucker-Drob and Salthouse 2009) and together with academic achievement scores (Marshalek et al. 1983; McGrew 2012; McGrew et al. 2014; Snow et al. 1984). However, in the context of the thousands of factor-analytic studies used with intelligence and academic achievement scores, MDS has been applied rarely. 

MDS puts all of the variables (e.g., tests) in continuous, geometric space based on their intercorrelations, and MDS “maps” of variables are created (Borg and Groenen 2005; Tucker-Drob and Salthouse 2009). Highly related scores are spatially closer in MDS maps, facilitating the interpretation of shared characteristics. For example, in previous MDS research with intelligence test constructs, verbal comprehension tests clustered together in one area of the map that was separate from other clusters of tests (Cohen et al. 2006; Marshalek et al. 1983; Meyer and Reynolds 2018).

One advantage of MDS is that it allows shared test characteristics to emerge because the tests are displayed in continuous space, and all variables remain in the model for interpretation instead of being reduced to a smaller number of variables as in principal components analysis or factor analysis. Objects in the MDS map can “differ along many dimensions simultaneously” (Snow et al. 1984, p. 89). Another advantage of MDS is that it does not impose as many expectations as in factor analysis (Tucker-Drob and Salthouse 2009).

### 1.3. MDS with Intelligence and Academic Achievement

Intelligence and academic achievement test score correlations can be analyzed together with MDS procedures. Pairs of tests with higher correlations are expected to be closer to each other in the MDS configuration. Additionally, the center of the map is the shortest distance from all other points, so tests with the strongest correlations with all other tests are expected to be in the center of the MDS map. Tests that do not correlate highly with all other tests are expected to be farther from the center of the MDS configuration, typically in clusters of highly related tests (Marshalek et al. 1983; McGrew et al. 2014). Although historically the focus has been on test characteristics and not latent abilities, conceptually, tests in the center are at-times more “*g*-related” and tests are often grouped by CHC broad abilities (Meyer and Reynolds 2018). Therefore, studies have indicated that both test complexity and test content are mapped, but the interpretations often parallel those from factor analysis and CHC theory (Marshalek et al. 1983; Guttman and Levy 1991). 

Shared test content (e.g., verbal tests) or CHC abilities are often useful in describing the organization of tests in clusters or regions of an MDS configuration (Marshalek et al. 1983; McGrew et al. 2014; Meyer and Reynolds 2018). For example, fluid reasoning tests may be clustered together, comprehension-knowledge tests clustered together, and visual processing tests clustered together. Within those CHC clusters, however, tests with higher *g*-loadings are often located closer to the center of the map forming what is called a “radex”.

Due to this arrangement in space, researchers have also interpreted test complexity as another dimension observable from MDS analysis of intelligence test scores. A typical pattern of MDS analysis of intelligence tests alone is for the most complex tests (higher-order thinking) to be near the center of the map (Marshalek et al. 1983; Tucker-Drob and Salthouse 2009), even though they differ in content. Tests near the center also often have the highest *g*-loadings, although this is not always the case (e.g., McGrew et al. 2014). For example, Marshalek et al. (1983) found fluid reasoning tests were closest to the center of the map–and these tests were considered the most cognitively complex tests. Comprehension-knowledge and visual processing tests were in the intermediate range of complexity. Memory and speed tests were farthest from the center of the MDS map. Therefore, the map seemed to place complex tests in the center. They described the continuum radiating out from the center as going from complex-general to simple-specific. Notably, however, the arrangement of the types of tests that radiate from the center also appear to be associated with the magnitudes of correlations between the latent CHC abilities and the *g* factor-fluid reasoning is the strongest, followed by comprehension-knowledge, visual processing, memory, and then processing speed (Carroll 1993). Likewise, intelligence test scores are often interpreted via CHC theory, with a focus more on composites such as the IQ as an indicator of *g* and broad indexes as indicators of CHC broad abilities (e.g., top down).

Academic achievement test data, on the other hand, are often interpreted more from the bottom up, and based on shared content. For example, rather than general reading, the more basic task of word reading or nonword reading scores are interpreted first before considering general reading ability, which is to some extent dependent on basic reading skills (and more of an emergent construct). Academic achievement test data also provide a clearer example of how both test content and complexity dimensions may appear in an MDS configuration. For example, reading tests should be located separately from other academic achievement domains (e.g., mathematics) and radiate from the center in a straight line. Reading comprehension (a complex reading task) should be closest to the center, and word recognition (the least complex reading task) should be farthest from the center. Reading fluency (intermediate complexity) would be located in the middle along an imaginary straight line connecting the reading comprehension and word recognition tests.

Most MDS research has been conducted with intelligence test scores. We are not aware of MDS studies with achievement tests only. MDS research with intelligence and academic achievement test scores analyzed together is limited to a few studies conducted 35 years ago, analysis of Woodcock-Johnson Psycho-Educational Battery—Revised and Woodcock-Johnson III data (McGrew 2012), and research reported in the Woodcock-Johnson IV test manual (McGrew et al. 2014). 

McGrew et al. (2014) divided the Woodcock-Johnson IV Tests of Cognitive Abilities, Tests of Academic Achievement, and Tests of Oral Language normative data into age-based subsamples for MDS analysis. At least six notable general findings emerged from the analysis. First, CHC abilities described test location better than test content. For example, the Pair Cancellation test, a processing speed test that contains visual stimuli, was closer to processing speed tests than it was to visual processing tests. Second, “regions” emerged that often consisted of two or more CHC abilities: auditory-linguistic, figural-visual, reading-writing, quantitative-numeric, and speed-fluency regions. Shared components in these regions better explained test location than content—for example, Writing Fluency, Word Reading Fluency, and Math Facts Fluency tests clustered within the speed-fluency region, next to the reading-writing and quantitative-math regions. The shared speed component better explained the location of fluency tests not their content. Third, academic achievement tests were mostly separate from intelligence tests. In some instances with the achievement tests, tests were organized with higher-order thinking tests located closer to the center of the MDS map (e.g., Applied Problems math test) and simpler tests farther from the center (e.g., Calculation basic math test). Fourth, achievement tests clustered closer to some cognitive test clusters than others. Reading and writing tests were closer to comprehension-knowledge and auditory processing CHC clusters (in the auditory-linguistic region), and math tests were closer to working memory and visual processing CHC clusters (in the figural-visual region). Fifth, tests did not all radiate outward from complex to simple in exact order by complexity as indicated by *g*-loadings (e.g., McGrew et al. 2014). Sixth, there were slight changes across age groups. A general memory region was found in the 6–8 age group, but not in others—theoretically, because the relations among cognitive abilities and achievement constructs do change with age, some age-related changes may be expected (e.g., Hajovsky et al. 2014). In light of these findings, MDS research with measures other than the Woodcock-Johnson tests is needed. Were they findings specific to the Woodcock tests? Or are these more generalizable findings? 

### 1.4. Facet Theory

MDS provides an opportunity to interpret test score relations in content (Guttman’s mode of communication) and complexity (Guttman’s rule inference) simultaneously. MDS is often associated; however, with Guttman’s facet theory that organizes and defines observations, such as those elicited during intelligence assessment. His facet theory also included response mode (Guttman’s mode of expression), which refers to how examinees respond to test items: oral, manual manipulation of materials (pegs, tiles, or blocks), and written (Guttman and Levy 1991). Response mode has been a useful explanation to test scores when three dimensional MDS maps are formed with intelligence scores (Cohen et al. 2006). Response processes are also considered important when evaluating validity evidence (American Educational Research Association et al. 2014), but rarely submitted to empirical analysis. Here, we wanted to consider response processes in our analysis. Hence, test scores may be correlated due to similar complexity (or how closely they related to psychometric g), content (or latent CHC broad abilities), and response processes. These three dimensions have emerged from a combination of Guttman’s facet theory, CHC theory, and research using MDS to map intelligence score correlations (Cohen et al. 2006; Marshalek et al. 1983). We wanted to investigate if they emerged in some way when including data from intelligence and achievement tests.

### 1.5. Purpose of the Study 

The purpose of this study was to use MDS to analyze correlations among Wechsler cognitive and achievement tests and among Kaufman cognitive and achievement tests to better understand the relations among the scores. We did so for several reasons. 

First, we wanted to use an alternative multivariate method to analyze intelligence and academic achievement test score correlations in combination. The majority of research has used factor analysis or some other form of structural equation modeling. Although MDS is used rarely, according to Snow et al. (1984, p. 88), because MDS “stays close to the original measures, requires minimal assumptions, and provides a simple representation, it is the method of choice when only one method is used.” Limiting theory and findings to certain statistical methods, such as factor analysis, may obscure nuance and limit new and important findings, especially when assumptions and choices used in new research depend on previous findings based on the same method. Do important findings emerge from analyzing these data with MDS? Even if the analysis produces results that are similar to those from factor analysis, similar findings with alternative methods only bolster confidence in the previous findings. 

Second, relatively few studies have used MDS to analyze intelligence test score correlations. Fewer have used MDS to analyze intelligence and academic achievement score correlations together. Explanations of findings from these studies have paralleled those from hierarchical factor analysis (Marshalek et al. 1983; Meyer and Reynolds 2018), such that CHC theory rather than test content may be used to explain clusters of tests in MDS space and how tests or clusters of tests radiate out from the center of MDS space (e.g., *g*-loadings are associated with a complexity dimension [fluid reasoning tests in the center of the map]). Those studies focused on intelligence test data, however. What happens when achievement tests are included? For example, does CHC theory still help to interpret findings? Or does test content also need to be considered.

Third, response processes are important aspects of test score validity. They are rarely evaluated using empirical methods, however. In addition to complexity and content (or their CHC parallels), do response processes help to understand correlations among intelligence and achievement tests when they are placed in multivariate space? If so, it may help with test score interpretation.

Fourth, revisions of popular tests, such as the Wechsler and Kaufman tests (Benson et al. 2019), have not been included in MDS studies with intelligence and achievement scores at all. Are findings related to the Woodcock tests generalizable? Specifically, McGrew et al.’ (2014) analysis also suggested larger auditory-linguistic, figural-visual, reading-writing, quantitative-numeric, and speed-fluency conceptual regions are useful in describing the scores in space. We wanted to test the reliability and viability of those regions with data from different test batteries. They are useful conceptual categories, but they would be much less interesting and useful if they do not emerge in data outside of the Woodcock tests. 

Last, we wanted to analyze whether these findings change across different developmental levels. On the one hand, analyzing data across ages or grades tests the reliability of the findings. Findings are not expected to drastically change across developmental levels so the findings should generally be consistent across the ages (Reynolds et al. 2007; Reynolds and Keith 2017). On the other hand, McGrew et al. (2014) found slight developmental changes and other research shows the relations between cognitive and academic may change with age—for example, comprehension knowledge is more highly associated with reading comprehension in adolescents than it is in younger children (Hajovsky et al. 2014). Therefore, because the Kaufman data were from a large sample, we divided the data into different grade level groups for analysis.To achieve our purpose, we asked the following research questions. Each question is accompanied by initial hypotheses. Each question applies to the different grade groups.

- Are complex tests in the center of the MDS configuration with less complex tests farther from the center of the MDS configuration?
- Intelligence and academic achievement tests of higher complexity were predicted to be near the center of the configuration and tests of lower complexity were predicted to be on the periphery (Marshalek et al. 1983). However, tests were not necessarily expected to all radiate outward from complex to simple tests in exact order by complexity as indicated by *g*-loadings (e.g., McGrew et al. 2014). Are intelligence tests and academic achievement tests clustered by CHC ability and academic content, respectively?
- Ga, Gc, Gv, Gf, and Gsm or Gwm tests were expected to cluster by CHC ability, and reading, writing, math, and oral language tests were expected to cluster by academic achievement area. Certain regions of academic achievement tests were predicted to align more closely with CHC ability factors. Reading and writing tests were predicted to be close to the Gc, Ga, and oral language tests. Math tests were predicted to be closer to the Gsm or Gwm, Gv, and Gf clusters.
- Are tests organized into auditory-linguistic, figural-visual, reading-writing, quantitative-numeric, and speed-fluency regions?
- Auditory-linguistic, figural-visual, reading-writing, quantitative-numeric, and speed-fluency regions were investigated in this study (McGrew et al. 2014). Gc tests, Ga tests, and oral language tests were predicted to cluster together with each other within an auditory-linguistic region. Reading and writing tests were predicted to be located in a reading-writing region. Gf tests were predicted to be in figural-visual or quantitative-numeric regions. Gv tests were predicted to be in a figural-visual region. Gsm or Gwm tests were predicted to be in the region that corresponded to the figural or numeric content (i.e., tests with pictures in the figural-visual region and tests with numbers in the quantitative-numeric region). Glr tests were not expected to be in just one region or in the same region of every configuration (McGrew et al. 2014).

## 2. Materials and Methods

### 2.1. Participants

#### 2.1.1. Wechsler Sample Participants

Data for the MDS with the WISC-V and WIAT-III were correlations derived from participant scores in a WISC-V validity study (see Wechsler 2014) with an average testing interval a little over two weeks (*M* = 15.5, *SD* = 14.37). Data from 181 English-speaking children and adolescents between the ages of 6 and 16 were used. Demographics are in Table 1. Demographics were mostly similar to the WISC-V norming sample and therefore similar to the U.S. population in 2012.

#### 2.1.2. Kaufman Sample Participants

MDS with the KABC-II and KTEA-II together were based on correlations derived from participant scores who were administered the tests as part of co-norming the tests, with a testing interval of 0 to 104 days, or an average of 8 days (Kaufman and Kaufman 2004). MDS was conducted by different grade levels, and the correlations matrices for grades 1–3 (*n* = 592), grades 4–6 (*n* = 558), grades 7–9 (*n* = 566), and grades 10–12 (*n* = 401) formed four subsamples. These four subsamples were chosen because of possible developmental shifts with these data in other research (Hajovsky et al. 2014). Demographics are shown in Table 2. Subsample demographics were mostly similar to those of the entire norming sample.

### 2.2. Measures

#### 2.2.1. WISC-V and WIAT-III

The Wechsler Intelligence Scale for Children-Fifth Edition (WISC-V; Wechsler 2014) is an individually administered assessment of intelligence for children and adolescents between 6 years and 16 years, 11 months. The WISC-V provides ten primary subtest scores, six secondary subtest scores, and five complementary subtests scores. Seven of the primary subtest scores combine to form the Full-Scale IQ (FSIQ), an index of general intelligence. Pairs of the primary subtest scores combine to form five primary indexes (Verbal Comprehension, Visual-Spatial, Fluid Reasoning, Working Memory, and Processing Speed). Twenty-one of 21 subtests were included in the study. The Wechsler Individual Achievement Test, Third Edition (WIAT-III; Breaux 2009) is an individually administered measure of academic achievement for children and adolescents between the ages of 4 and 51 years, (Breaux 2009). Alphabet Writing Fluency and Early Reading Skills were excluded from analysis because they were only administered to examinees in Grade 3 or below (*N* = 44) so 14 of 16 subtests were included here. 

#### 2.2.2. KABC-II and KTEA-II

The Kaufman Assessment Battery for Children, Second Edition (KABC-II; Kaufman and Kaufman 2004) is an individually administered assessment of children and adolescents’ processing and cognitive abilities between 3 years and 18 years, 11 months. The KABC-II provides a composite as an estimate of general intelligence and multiple CHC indexes. There were 15 of 18 subtests included in the study. The Kaufman Test of Educational Achievement, Second Edition (KTEA-II; Kaufman 2004) is an individually administered measure of academic achievement for children and adolescents between the ages of 4 years, 6 months and 25 years, 11 months (Kaufman 2004). There were 14 or 16 of 16 subtests included in the study depending on the age range.

### 2.3. Data Preparation Prior to MDS Analysis

MDS was conducted separately for each dataset: one with WISC-V and WIAT-III, and one with each of the four KABC-II and KTEA-IIs. Prior to MDS, symmetrical correlation matrices were created from bivariate correlations between subtest (simply “test” from now on) standard scores. From the WISC-V and WIAT-III data, 1.5% of the scores were missing, mostly from Math Fluency Multiplication and Essay Composition. From the Kaufman Grades 1–3 data (*n* = 592), 5.3% was missing mostly from Decoding Fluency and Word Recognition Fluency. From the Kaufman Grades 4–6 data (*n* = 558), Kaufman Grades 7–9 data (*n* = 566), and Kaufman Grades 10–12 data (*n* = 401), less than 1% was missing from each. Pairwise deletion was used for calculating correlations and not considered a problem based on such small amounts of missing data (Graham 2009).

Dissimilarity matrices were constructed from the correlation matrices. The formula $\sqrt{1-r}$ was used to convert Pearson correlations for each pair of tests to dissimilarities. Each dissimilarity matrix was submitted to MDS procedures. 

### 2.4. MDS Analysis

Five symmetrical dissimilarity matrices were inputted separately to the MDS algorithm. Initial model specifications included initial configuration (the starting location for each object in the matrix from which the algorithm produces iterations of configurations), type of transformation (whether dissimilarities are treated as interval- or rank-level data), and number of dimensions to be represented in the configuration. Configuration of objects were plotted for visual analysis and interpretation. Statistics and visualization were conducted with R 4.0.4 (R Core Team 2021), smacof (Mair et al. 2021), ggplot2 (Wickham 2016), and rgl (Adler and Murdoch 2021) packages. The output from the MDS smacof package (Mair et al. 2021) included the configuration of objects (coordinates for each point in the specified number of dimensions) and an estimate of model misfit between the dissimilarity matrix and MDS configuration, called stress. Different specifications for the model affect fit and were compared to select the best MDS model for each matrix.

#### 2.4.1. Model Selection

Model selection was based on fit and interpretability. Four different models were estimated for each matrix: (1) interval transformation in two dimensions; (2) interval transformation in three dimensions; (3) ordinal transformation in two dimensions; and (4) ordinal transformation in three dimensions. Stress is a loss function that helps with deciding or confirming model specifics from the MDS procedure. Perfect fit results in 0 stress. The maximum stress is 1 (Cohen et al. 2006). Global stress was considered for absolute fit. Kruskal’s (1964) guidelines were used as a starting point: .20 is poor, .10 is fair, .05 is good, and .025 is excellent. Stress also increases when the number of objects in MDS analysis increases, so those are not considered cutoff values. The rules for acceptable stress may be too strict for MDS with the large number of objects in each matrix in this study (Mair et al. 2016). Thus, stress from random permutations of dissimilarities were also compared to model stress. The null hypothesis is that stress in the MDS configuration from the study data is as high as or higher than stress from MDS of random dissimilarity matrices. If the null hypothesis is rejected, stress in the model is likely lower than stress in the random dissimilarity matrices—indicating acceptable stress from an absolute standpoint.

Global stress was also used to compare the relative fit of two- or three-dimensional models. The contributions of individual points to global stress (stress-per-point) were considered. In some cases a few points may account for most of the stress (Borg et al. 2018).

#### 2.4.2. Preparation for Interpretation

Once each final model was selected, the MDS configurations were plotted using ggplot2 (Wickham 2016), and rgl (Adler and Murdoch 2021) packages in R. Test abbreviations and *g*-loadings were used to label tests in the MDS configuration scatterplots. In one version of each configuration scatterplot, tests were color-coded by complexity and a sphere was added to indicate the center point. In another version of the configuration scatterplot, tests were color-coded by CHC ability factor or academic achievement domain, and lines connected tests from the same CHC ability factor or academic achievement domain to ease interpretation. Last, WISC-V and WIAT-III tests were color-coded by content or by response mode to help with visual analysis and explore similarities in content and response modes. 

To support visual inspections in answering questions about complexity, the center point of each MDS configuration was calculated and depicted as a black sphere. The center point was defined as the mean of each dimension $\left(\bar{x},\bar{y},\bar{z}\right)$. The distance from each subtest to the center point was calculated. Where the distance between $P_{1}=\left(x_{1},y_{1},z_{1}\right)$ and $P_{2}=\left(x_{2},y_{2},z_{2}\right)$ is calculated by $d\left(P_{1},P_{2}\right)=\sqrt{{\left(x_{2}-x_{1}\right)}^{2}+{\left(y_{2}-y_{1}\right)}^{2}+{\left(z_{2}-z_{1}\right)}^{2}}$.

Last, Spearman’s rank-order correlation was calculated for intelligence test *g*-loadings and distance from the center point of the MDS configuration to help quantify the organization of intelligence tests in the MDS map in terms of complexity.

## 3. Results

### 3.1. Preliminary Analysis and Model Selection

Global stress for the MDS configurations across the five datasets ranged from .06 (good) to .29 (poor) depending on the matrix, type of transformation, and number of dimensions. Table 3 shows stress values for ordinal and interval transformations in two and three dimensions. Stress values in all MDS configurations were lower than the random permutations. MDS models fit the data in the dissimilarity matrices better than a configuration of random dissimilarities and were minimally acceptable in terms of absolute fit. Stress values of different model configurations were compared for relative fit. In terms of relative fit, ordinal, three-dimensional configurations fit the data best. Ordinal fit better in general because rank order of distances is preserved instead of the relative size of distances between objects, and rank order is simpler to remain consistent between the input and MDS configuration. 

Last, stress-per-point values of three-dimensional MDS models were examined to identify tests that accounted for much more stress than other points. All tests were retained in the MDS configurations because the three-dimensional stress values were fair or good as is. 

### 3.2. Primary Analyses

#### 3.2.1. WISC-V and WIAT-III Model Results

The ordinal, three-dimensional model was selected for the WISC-V and WIAT-III data. The three-dimensional MDS configuration was plotted. The following research questions were answered based on visual analysis of the three-dimensional scatterplot (with different color-coding versions) and calculations of distances from the configuration center.

1. Are complex tests in the center of the MDS configuration with less complex tests farther from the center of the MDS configuration?

**Figure 1:**
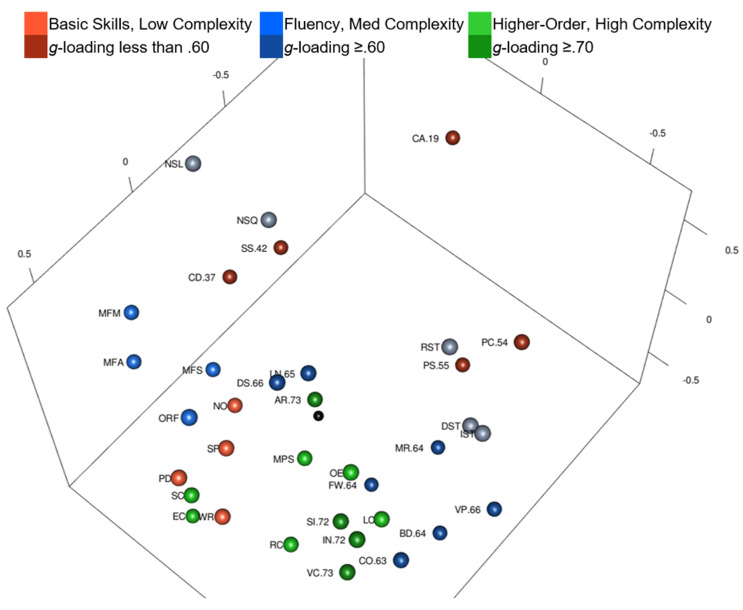
WISC-V and WIAT-III 3D MDS Configuration, Color-Coded by Complexity.

Yes and no. Intelligence and academic achievement tests, color-coded by complexity, are shown in Figure 1 (Figure 1 is also available as an interactive 3D graphic in the Supplemental Figures). Intelligence test *g*-loadings are in the test labels, when available; intelligence tests with unknown *g*-loadings are gray. The black sphere in Figure 1 is the center of the MDS configuration. Complex tests are not all in the center of the MDS map (i.e., green tests in Figure 1 are not all in the center of the map). When rotating the interactive 3D configuration, Arithmetic (highest *g*-loading of .73) is closest to the center of the MDS configuration followed by Math Problem Solving, but other complex tests (e.g., Essay Composition) are as far away as basic skills or fluency tests. Other tests with *g*-loadings higher than .70 (Vocabulary *g*-loading of .73 and Information *g*-loading of .72) are on the periphery with tests of lower *g*-loadings. By visual inspection alone, tests do not radiate outward with the highest complexity in the center and the lowest complexity tests on the periphery.

Tests in Table 4 are in order by distance from the WISC-V and WIAT-III MDS configuration center point. In the ranked list, Arithmetic is closest to the configuration center (.09 units from center) followed by Math Problem Solving (.24 units from center). Cancellation is the farthest from the configuration center (1.33 units from center). Intelligence and academic achievement tests are dispersed throughout the rankings (i.e., the intelligence tests are not all at the top or bottom of the rank order). Several high complexity intelligence and academic achievement tests are within .6 units from the center, but Essay Composition, Matrix Reasoning, Block Design, and other higher complexity tests are as far from the center as lower complexity intelligence and academic achievement tests. A Spearman’s rank-order correlation was calculated to assess the relation between intelligence test complexity (*g*-loadings when available) and distance from the MDS configuration center. There was a statistically significant, strong negative correlation between intelligence test *g*-loadings and distance from the configuration center, *r*s(14) = −.715, *p* < .01. Intelligence tests with higher *g*-loadings were more likely to be closer to the center of the configuration, but those are only based on intelligence tests with *g*-loadings.

2. Are intelligence tests and academic achievement tests clustered by CHC ability and academic content, respectively?

**Figure 2:**
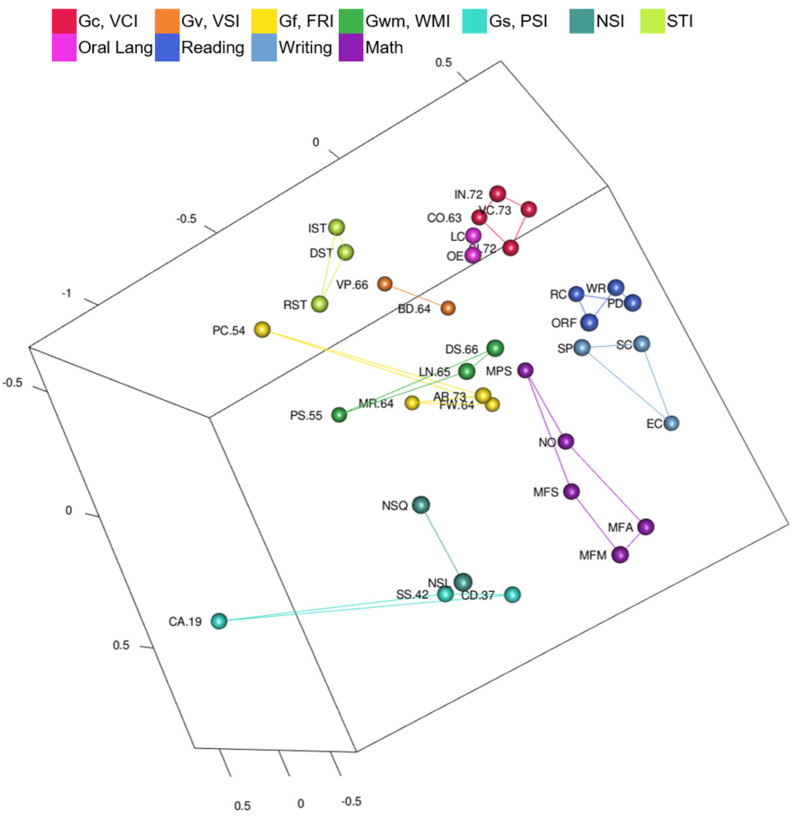
WISC-V and WIAT-III 3D MDS Configuration CHC and Academic Clusters.

Yes. WISC-V and WIAT-III MDS CHC ability and academic domain clusters are visible in Figure 2 (Figure 2 is also available as an interactive 3D graphic in the Supplemental Figures). Tests are color-coded by CHC ability or academic achievement domain and lines connect the clusters of tests. Clusters are generally separate (e.g., there are no math tests inside of the reading cluster) and clusters of tests are mostly separated by their different color-coding. Reading, writing, math tests in the WISC-V and WIAT-III 3D MDS configuration are in a separate wedge from intelligence tests (on the right-hand side in Figure 2). The writing cluster is between the reading cluster and the math cluster. The oral language cluster is separate from the other academic achievement areas, and much closer to the Gc cluster as shown in Figure 2. Math Problem Solving is closer than reading tests to the Gc cluster. Math tests are closer than reading and writing tests to the Gf cluster. The Math Problem Solving test involves similar questions and skills as the Arithmetic (Gf) test and requires higher-order thinking and problem solving like Arithmetic and the other fluid reasoning tests.

Even though the CHC and academic achievement clusters do not overlap (e.g., there is not a Gwm test within the cluster of Gc tests), some of the tests are near other clusters. Arithmetic is part of the Gf cluster, but it is located near the math cluster. Picture Span is part of the Gwm cluster, but it is located near the Picture Concepts test, a test with similar picture content (instead of letter and numeric content like the other Gwm tests). Oral Reading Fluency is in the reading cluster, but it is located near Digit Span and Letter-Number Sequencing Gwm tests. Gs and NSI clusters (lower left in Figure 2) are isolated from other clusters. Those tests have the lowest correlations with other tests. Lastly, Gf and Gv tests associated with primary WISC-V indexes were in separate clusters in the WISC-V and WIAT-III map.

3. Are tests organized into auditory-linguistic, figural-visual, reading-writing, quantitative-numeric, and speed-fluency regions?

**Figure 3:**
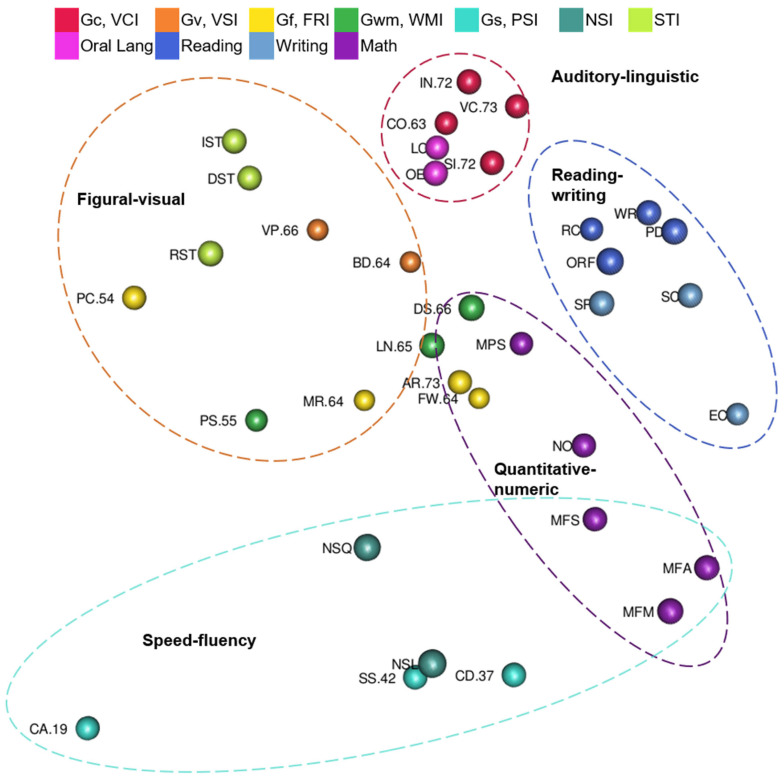
WISC-V and WIAT-III 3D MDS Configuration Regions.

Yes, though some regions overlap. Tests are color-coded by CHC ability or academic achievement domain, and dashed ovals highlight the auditory-linguistic, figural-visual, reading-writing, quantitative-numeric, and speed-fluency regions in Figure 3. The auditory-linguistic region includes Gc and oral language tests. The reading-writing region includes reading and writing tests. The quantitative-numeric region includes math achievement tests, memory tests with number stimuli, and fluid reasoning tests in which examinees *solve* novel math problems. The figural-visual region includes tests with pictures or other visual stimuli. The speed-fluency region includes processing speed, rapid naming, and math fluency tests. Math fluency tests are in the overlap between quantitative-numeric and speed-fluency regions.

#### 3.2.2. Kaufman Grades 4–6 Model Results

Only one Kaufman model was included due to space limitations. Similarities and differences across grade bands, however, will be discussed. See the Supplementary File for three-dimensional figures of the other Kaufman models. The ordinal, three-dimensional model was selected for the Kaufman Grades 4–6 data. The three-dimensional MDS configuration was plotted. The following research questions were answered based on visual analysis of the three-dimensional scatterplot (with different color-coding versions) and calculations of distances from the configuration center.

1. Are complex tests in the center of the MDS configuration with less complex tests farther from the center of the MDS configuration?

**Figure 4:**
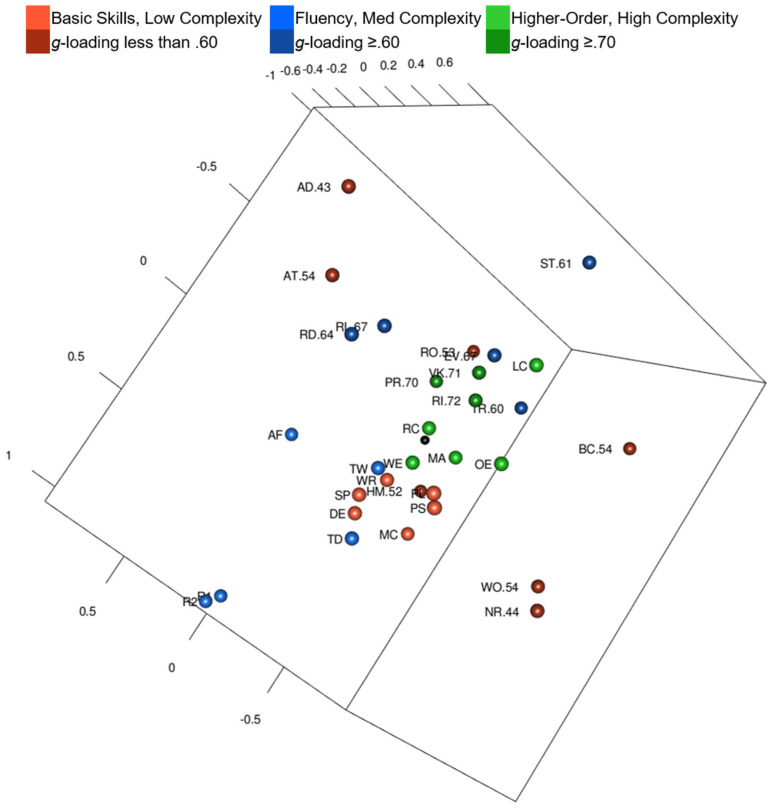
Kaufman Grades 4–6 3D MDS Configuration, Color-Coded by Complexity. Kaufman Grades 4–6 3D MDS Configuration Static View, Color-Coded by Complexity.

Yes and no. Intelligence and academic achievement tests are color-coded by complexity in Figure 4 (Figure 4 is also available as an interactive 3D graphic in the Supplemental Figures). Intelligence tests’ *g*-loadings are in the test labels, when available. The black sphere in Figure 4 is the center of the MDS configuration. Three complex academic achievement tests are closest to the black sphere: Reading Comprehension, Written Expression, and Math Concepts & Applications. Letter & Word Recognition (a low complexity test) is also close to the center. 

Tests in Table 5 are in the order by distance from the Kaufman Grades 4–6 MDS configuration’s center point. Reading Comprehension (.05 units from center), Math Concepts & Applications (.18 units from center), and Written Expression (.21 units from center) are closest to center. Riddles (.72) and Verbal Knowledge (.71) are the tests with the highest *g*-loadings; they are the intelligence tests closest to center. There was a statistically significant, strong negative correlation between intelligence test *g*-loadings and distance from center, *r*s(13) = −.842, *p* < .01. Intelligence tests with higher *g*-loadings were more likely to be closer to center than intelligence tests with lower ones. Tests with *g*-loadings ≥ .70 are closest to the center of the configuration, followed by tests with *g*-loadings > .61, and then tests with *g*-loadings ≤ .61. Intelligence tests are organized with more complex tests closer to center and less complex tests around the periphery, but again, seven out the ten tests closest to the center are achievement tests and not intelligence tests.

2. Are intelligence tests and academic achievement tests clustered by CHC ability and academic content, respectively?

**Figure 5:**
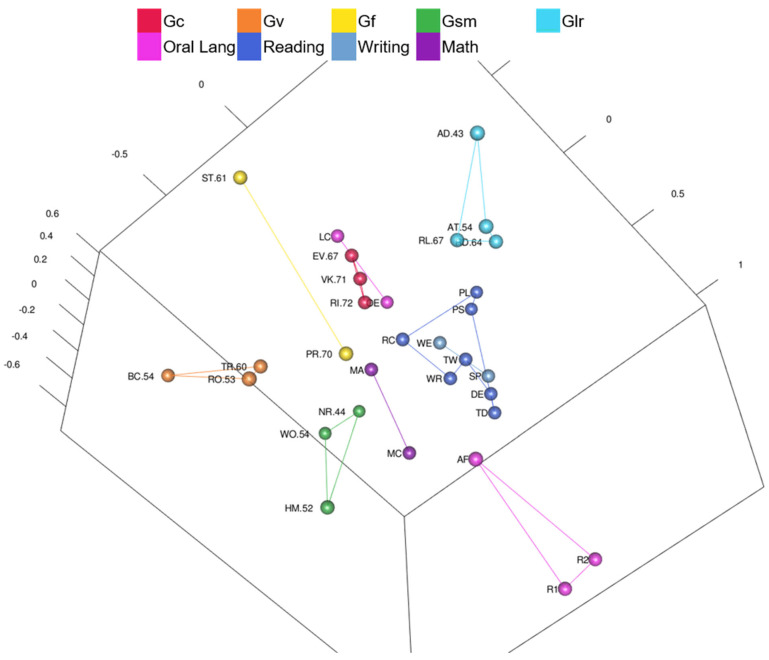
Kaufman Grades 4–6 3D MDS Configuration CHC and Academic Clusters.

Yes. Kaufman Grades 4–6 CHC ability and academic domain clusters are visible in in Figure 5 (Figure 5 is also available as an interactive 3D graphic in the Supplemental Figures). Tests are color-coded by CHC ability or academic achievement domain, and lines connect the clusters of tests. The writing cluster is inside of the reading cluster. The reading-writing cluster is near math academic achievement tests. Oral language tests are split into two clusters on either side of academic achievement test clusters. Oral Expression and Listening Comprehension are next to the Gc cluster. Naming Facility and Associational Fluency are on the other side of the MDS map. Math Concepts & Applications is closer to Gf tests, but Math Computation is not closer to Gf tests than are the reading tests. Most reading tests, except Reading Comprehension, are farther from Gc than Math Concepts & Applications.

3. Are tests organized into auditory-linguistic, figural-visual, reading-writing, quantitative-numeric, and speed-fluency regions?

**Figure 6:**
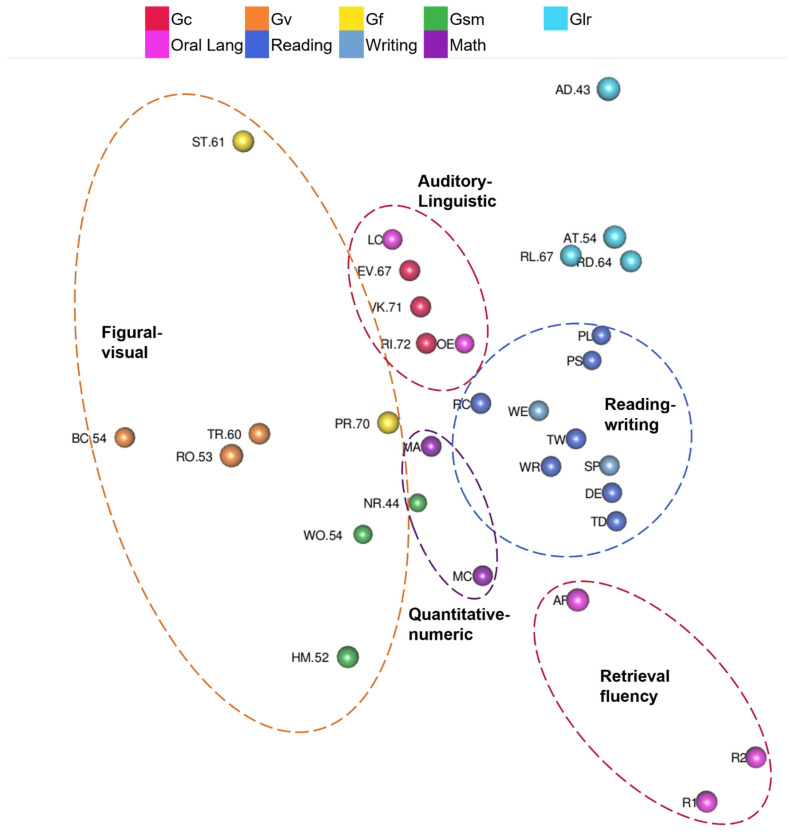
Kaufman Grades 4–6 3D MDS Configuration Regions.

Yes, but not speed-fluency. Tests are color-coded by CHC ability or academic achievement domain, and dashed ovals highlight the auditory-linguistic, figural-visual, reading-writing, and quantitative-numeric regions in Figure 6. Naming Facility tests and Associational Fluency were not in the auditory-linguistic region with Oral Expression and Listening Comprehension tests. Thus, those tests formed a small retrieval fluency region. The reading-writing region includes reading and writing tests. The two math academic achievement tests are near the reading-writing region and form the quantitative-numeric region. The figural-visual region includes tests with pictures or other visual stimuli. Tests in the figural-visual region are spread out more than the tests in other regions of the configuration.

### 3.3. Secondary Analyses

#### 3.3.1. Kaufman Grade Groups 

MDS results were mostly similar across grade groups, but there were some visible differences. Grade groups were similar in that among intelligence tests, those with higher *g*-loadings were closer to the center of the MDS configuration. They were also similar in that the tests closest to the center were more likely to be achievement tests than intelligence tests. The pattern of lower complexity tests radiating outward toward the periphery of the configuration was not as obviously visible. For example, Letter & Word Recognition was second closest to the center of the Grades 10–12 MDS configuration. 

Another consistent finding was that Gc, reading, writing, and math clusters tended to be in the center of MDS configurations for all grade groups. One potential developmentally related finding, however, was that Listening Comprehension and Oral Expression were closer to the center with the previously mentioned clusters at the upper grade levels (Grades 7–9 and Grades 10–12).

Additionally, the 3D MDS configuration regions were similar across grade groups. The maps all included clearly delineated auditory-linguistic, figural-visual, reading-writing, and quantitative-numeric regions. 

One potential developmental finding was related to where fluency tests from the oral language composites were located on the maps. In Grades 1–3 and 4–6, these fluency tests were on the opposite side of the MDS configuration from the other auditory-linguistic tests. In Grades 7–9 and 10–12, however, these fluency tests were on the same side of the MDS configuration as other auditory-linguistic tests. 

One other notable difference was found, but it was only related to one grade group. In each grade group, Word Recognition Fluency and Decoding Fluency were much closer to reading tests (clustered by academic content instead of by fluency); however, both of these fluency tests were on the side of the reading cluster closest to the Associational Fluency and Naming Facility tests in the Grades 7–9 MDS configuration. There was only a memory region in the Kaufman Grades 10–12 configuration. In the other grade configurations, Glr and Gsm tests were on opposite sides of the configuration or very far from each other.

#### 3.3.2. WISC-V and WIAT-III Content and Response Modes

**Figure 7:**
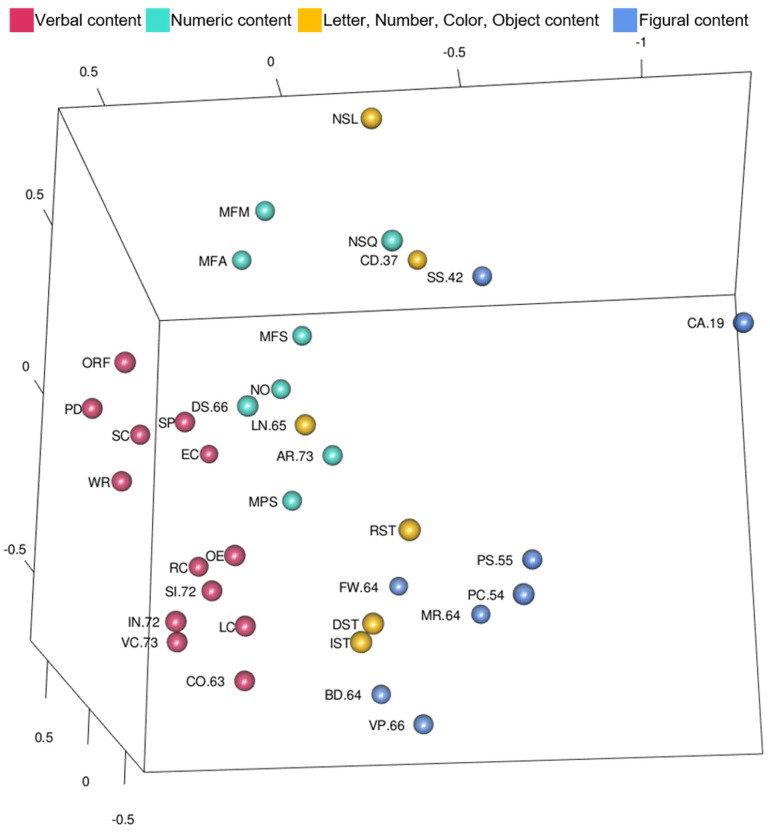
WISC-V and WIAT-III 3D MDS Configuration Static View, Color-Coded by Content.

Guttman and Levy (1991) interpreted two additional facets beyond complexity: content and response mode. In order to explore Guttman’s content facet in the WISC-V and WIAT-III model, tests were color-coded more broadly by the type of content or stimuli they include. This coding scheme is shown in Figure 7 (Figure 7 is also available as an interactive 3D graphic in the Supplemental Figures). Tests with verbal and figural content are clearly separated from each other and located in non-overlapping regions of the MDS configuration. Tests with numeric content also appear clustered closer together and in a region of their own. Some of the tests, like Letter-Number Sequencing or Coding, have combinations of different stimuli like symbols, letters, and numbers. The mixed stimuli tests are located throughout the MDS configuration, mostly between the numeric and figural content regions. Taken together and as shown clearly in Figure 7 the content facet is useful in analyzing larger patterns in the MDS configuration.

**Figure 8:**
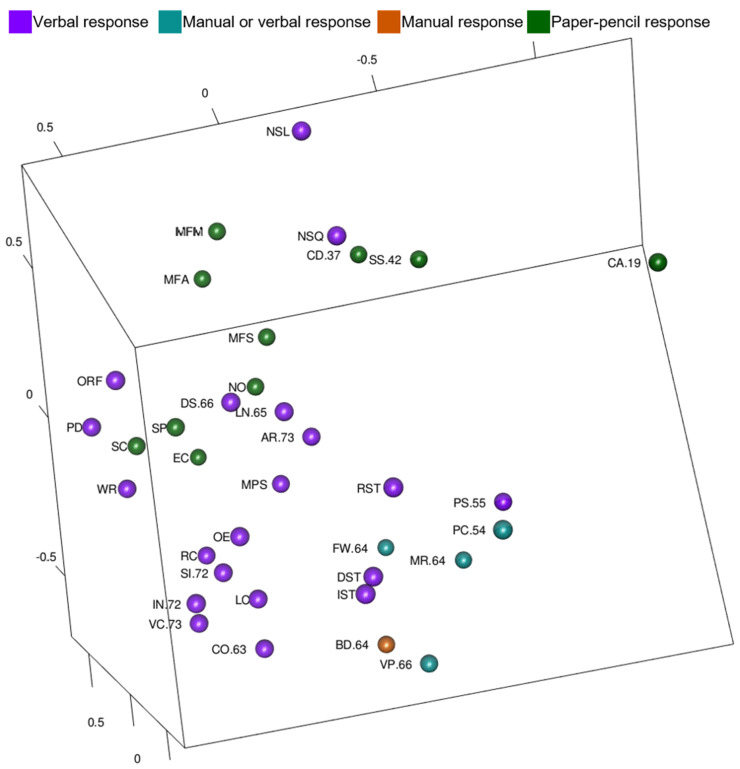
WISC-V and WIAT-III 3D MDS Configuration, Color-Coded by Response Mode.

In order to explore Guttman’s response mode facet in the WISC-V and WIAT-III model, tests were color-coded by the way an examinee responds and these are shown in Figure 8 (Figure 8 is also available as an interactive 3D graphic in the Supplemental Figures). Notably, tests with paper-pencil responses are mostly on one side of the MDS configuration and tests with verbal responses are mostly on the other side. Tests that examinees may respond verbally to or by pointing to are on the periphery of the MDS configuration (the lowest portion of Figure 8). Block Design is the only test in which a manual response (moving blocks) is required, and it is located near the tests with manual or verbal response options.

## 4. Discussion

The purpose of this study was to use MDS to analyze correlations among Wechsler cognitive and achievement tests and among Kaufman cognitive and achievement tests to better understand the relations among the scores. Three research questions were answered.

First, less complex academic achievement tests were in the center of the MDS configurations with complex academic achievement tests and intelligence tests with high *g*-loadings. Intelligence tests with high *g*-loadings were more likely to be near the center than intelligence tests with lower *g*-loadings. However, academic achievement tests were more likely to be near the center of the configuration than intelligence tests. Fluency tests were least likely to be near the center of the configuration. The finding made the complexity interpretation less clear and was an unexpected result. 

Second, intelligence and academic achievement tests were generally clustered by CHC ability and academic content, respectively. Reading, writing, complex oral language, and Gc tests were consistently clustered near each other. Complex math tests were closer to Gf tests than simpler math tests, but the math cluster was not always closer to the Gf tests than to Gc tests. CHC abilities were helpful in explaining the locations of intelligence constructs and academic achievement content areas were useful in explaining the academic area clusters.

Third, consistent with McGrew et al. (2014), tests were organized into auditory-linguistic, figural-visual, reading-writing, quantitative-numeric regions for all model results. Speed-fluency was more visible in WISC-V and WIAT-III results than the Kaufman results. However, Kaufman tests do not include processing speed tests, and in each Kaufman model there was a retrieval fluency region. At times, the regions were overlapping as some fluency tests clustered with speeded tests in a speed-fluency region and others clustered with tests of similar academic content. For example, math fluency tests were in the overlap between the quantitative-numeric and speed-fluency regions of the WISC-V and WIAT-III model. 

In addition to the research questions, most MDS results were similar across grade groups (complexity organization, Gc tests near the center, separation of complex oral language from oral fluency tests). There were, however, a few differences, with at least two possible developmental differences. Lastly, the Wechsler tests were organized by content and response processes.

Although several findings were expected, not all of our original hypotheses were confirmed. Regarding complexity, the addition of achievement tests appeared to cloud the interpretation of that dimension. As with prior research CHC classification works and confirmed McGrew et al.’s (2014) additional categorization of broader regions. Last, found that response processes may explain some of the correlations among tests. Additionally, some test specific findings emerged. We discuss these findings below.

### 4.1. Complexity

Complexity of a task refers to the number of cognitive processes involved, the importance of cognitive processes, attention and memory demands, and adaptation or executive functions required (Lohman and Lakin 2011). Here, for intelligence tests we also used *g*-loadings as indicators of complexity. For achievement tests, skill in more complex tasks tend to rely on skills measured in the less complex tasks. Previous studies with MDS of intelligence test score correlations have supported the radex model of intelligence, with complex tests near the center of two-dimensional or three-dimensions MDS configurations (Guttman and Levy 1991; Marshalek et al. 1983). 

#### 4.1.1. Wechsler Models

The WISC-V and WIAT-III model told a complicated story with no ending regarding complexity. Some findings were consistent with prior research. For example, in general, intelligence tests with higher *g*-loadings were closer to the center (Snow et al. 1984). The location of Arithmetic in the three-dimensional map with intelligence and achievement tests analyzed together was consistent with prior MDS research with the entire WISC-V standardization sample in two dimensions (Meyer and Reynolds 2018). Arithmetic, which also had the highest *g*-loading, was very near the configuration center. Arithmetic is classified as a Gf test according to the WISC-V manual (Wechsler 2014). With the complex math test (Math Problem Solving) being the next closest to the center, at first glance these findings appeared consistent with Marshalek et al.’ (1983) findings that showed Gf tests in the center of their MDS map of WAIS and other intelligence tests, with reading and math composites close by. A closer look, however, revealed that in the current study, the remaining Gf tests were some of the furthest from the center. This finding is not only inconsistent with Marshalek and colleagues’ findings, but also with the findings from factor analysis that *g* and Gf are statistically indistinguishable (Gustafsson 1984). That is, “*g*” in MDS is associated with the center of the MDS map. In addition to Arithmetic, other tests that were closest to the center in the current study were two math tests (Numerical Operations and Math Problem Solving), two working memory tests (Digit Span and Letter-Number Sequencing), and Spelling. Besides Arithmetic and Math Problem Solving, these other tests are not considered the most complex. These working memory test locations in the configuration are inconsistent with findings from previous research in which memory tests (Digit Span Forward, Digit Span Backward, Auditory Letter Span, and Visual Number Span) were the furthest away from the center (Marshalek et al. 1983). Here, complexity did not seem to be the reason these tests were closest to the center with WISC-V and WIAT-III tests. Besides Spelling, all of the tests in the center of the WISC-V and WIAT-III MDS configuration involve numbers, so that may explain why they were closer to each other, but it breaks from prior research (Cohen et al. 2006; Guttman and Levy 1991; Marshalek et al. 1983; Meyer and Reynolds 2018) in that something besides complexity is explaining why tests are located in the center of the model—almost appearing to be test content related. Returning to Arithmetic, the constructs measured by Arithmetic have been debated, although Gf, Gwm, and math reasoning have all been implicated, so it is notable that it was close to other Gwm and mathematics tests in this study (Keith and Reynolds 2010). 

The remaining WISC-V and WIAT-III tests also failed to show a pattern like the radex model with low complexity tests around the periphery and high complexity tests in the center. Instead, high and low complexity tests were intermixed in their distances from the center. For example, complex reading, oral language, writing, and Gc tests were farther away from the center even though these are considered some of the most complex tests. Although complexity has been described as a “modulating” facet that determines a test’s distance from the center in a MDS map (Guttman and Levy 1991, p. 97), the pattern of intelligence and achievement tests in combination and in relation to their distances from the center in this study almost seemed to arranged by content features. 

#### 4.1.2. Kaufman Models

The Kaufman MDS model organization by content seemed more apparent than organization by complexity. At the surface, the Kaufman MDS map appeared to follow findings related to complexity and *g*-loadings (Marshalek et al. 1983; Snow et al. 1984; Tucker-Drob and Salthouse 2009). Intelligence tests correlated almost perfectly with distance from the center of the configuration and *g*-loadings, but upon closer inspection, it was only intelligence tests relative to other intelligence tests in terms of *g*-loadings that were closest to the center. Overall, the achievement tests were closer (both complex and not complex) to the center. This finding is counter to Marshalek et al.’s (1983), but in that study, only academic achievement composites were included so less complex academic achievement tests could not be in the center of the configuration. In the Snow et al. (1984) MDS analysis with Thurstone’s (1938) ability data, none of the simple math tests (addition, subtraction, and multiplication) were in the center of the MDS map, but it is unknown whether these tests were simple and timed, like the fluency tests, or like the basic calculation tests in the present study. In the current study, basic math calculation and math problem solving tests were closer to the center of the map.

One clear feature of the Kaufman MDS maps was that reading, writing, math, and oral language comprehension tests were either near or intermediate distances from the center of the configuration. Across the grade bands of Kaufman MDS configurations, high complexity academic achievement tests (Math Concepts & Applications, Reading Comprehension, and Written Expression) were near the center. Gc tests were often near the center too. Less complex reading academic achievement tests from the KTEA-II were close to the center of the MDS configuration (e.g., Letter & Word Recognition was fourth from the center for Grades 1–3 and Grades 4–6, second from the center for Grades 7–9 and Grades 10–12). Nevertheless, exceptions to the radex organization, like Letter & Word Recognition and Numerical Operations, stand out when analyzing by visual inspection. The verbal centric Kaufman MDS configurations with Gf, Gv, Glr, and Gsm tests farther away from the Gc, reading, writing, and math tests called back to Vernon’s (1950) hierarchical model with verbal:educational and spatial:mechanical group factors and Cattell’s (1943) fluid intelligence and crystallized in intelligence. In this study, the verbal:educational tests (crystallized intelligence) were in the center of the Kaufman configurations and spatial:mechanical (fluid intelligence) tests were to the side and periphery of the models.

One take home from these findings may be that intelligence tests do tend to generally emanate outward from the center in a way that aligns with tests with higher *g*-loadings being closer to the center. However, when considering the realm of all tests measuring cognitive abilities and developed achievement areas, the complexity dimension is not as clear. Thus, although findings are generally consistent with prior studies with intelligence tests only, it is likely premature to consider that dimension as one that is directly related to the complexity of the task. 

### 4.2. CHC and Academic Clusters

CHC theory is framework of latent cognitive abilities (Keith and Reynolds 2010) and academic domains, like reading, writing, and quantitative knowledge. Though the interpretation of complexity was unclear, organization of subtests by CHC (Gc, Gv, Gf, Gwm or Gsm, Gs, if applicable, and Glr, if applicable) and academic domain (reading, writing, math, oral language) was very clear in all of the MDS plots. The findings in general appeared to be consistent with factor analytic evidence (Reynolds and Keith 2007; Reynolds and Keith 2017). 

It was helpful to examine the MDS results through the lens of CHC factor structure and the scoring structure of academic achievement tests to subdivide the geometric space. These clusters are a succinct way to summarize results and are useful for quickly finding tests in a visually dense representation like the 3D MDS configurations. CHC and academic clusters were very consistently aligned with CHC factors and academic domains. Some of the clusters, like WISC-V Gf and Gs clusters or the KABC-II Gv cluster were more spread out in geometric space, but the lines connecting CHC and academic clusters never made a messy web of lines. The clusters, except for writing and reading, were distinct from each other. 

Similar to McGrew et al. (2014) results, tests clustered by CHC and academic domain. Tests in the Wechsler and Kaufman CHC and academic domain clusters stayed together as predicted, with the exception of the oral language tests and oral fluency tests separating. In CHC parlance, however, oral retrieval fluency tests in the KTEA-3 (Kaufman et al. 2014) are known as tests of ideational fluency and rapid naming, the latter of which is also measured by the Naming Speed tasks in the WISC-V. Oral retrieval fluency tests from the KTEA-II were not close to the other oral language tests in the auditory-linguistic region, and seemed to form a separate retrieval fluency region. The retrieval fluency region was similar to the speed-fluency region found in the WISC-V and WIAT-III maps, except no tests of processing speed are included in the Kaufman tests. This fluency region was also similar to the retrieval fluency factor, Gr, recently identified as separate from learning efficiency (Jewsbury and Bowden 2016; Schneider and McGrew 2018). 

In addition to clear definition of the geometric space in terms of CHC abilities and academic domains, there were interesting findings related to the tests themselves. For example, the WISC-V scoring structure separates tests into Fluid Reasoning and Visual Spatial indexes (Wechsler 2014), even though WISC-V fluid reasoning tests require examinees to reason with visual content. The separation of Gv and Gf clusters in the WISC-V and WIAT-III map supported separate composites for the primary indexes. At the same time, shared visual content that contributes to Gv and Gf clusters being near each other in the map is worth considering more carefully (cf., Reynolds and Keith 2017).

In addition, WISC-V Picture Span was part of the Gwm cluster (and included on the Working Memory Index on the test), but it was located near the Picture Concepts test that has similar picture content (instead of letter and numeric content like the other Gwm tests). When Picture Span and Picture Concepts were analyzed in a two-dimensional MDS configuration in previous research, they were across from, and not next to, each other in the MDS map (Meyer and Reynolds 2018). The current study, however, included more dimensions and academic achievement tests. A potential explanation for different findings comes from Guttman’s content facet (shown in Figure 7). The inclusion of academic achievement tests in the WISC-V and WIAT-III 3D MDS configuration introduced several tests with verbal content (reading and writing tests with Gc and some oral language tests on the left side of Figure 7). There were also more tests with numeric content (to the right and above verbal content tests in Figure 7). Including WIAT-III scores did not contribute additional tests with primarily symbols, pictures, or figure content. It is possible that stronger correlations among tests with verbal content and among tests with numeric content allowed the correlations among tests with pictorial content to become more pronounced or visible in the configuration instead of being “pulled” into CHC ability clusters. Content features may be considered when interpreting scores from these measures.

Naming Speed Literacy and Naming Speed Quantity tests were new to the WISC-V and meant to be sensitive to specific learning disorder-reading and -mathematics, respectively (Wechsler 2014). These tests measure rapid automatic naming, another component process that is important for efficient and accurate reading (Norton and Wolf 2012). Visible in Figure 2 interactive 3D MDS configuration, Naming Speed tests from the WISC-V were on the same side of the configuration as Digit Span, Letter-Number Sequencing, and Oral Reading Fluency. Each of these tests require verbal responses, but they also likely measure one or more latent Gwm narrow abilities: auditory short-term storage, visual-spatial short-term storage, attentional control, and working memory capacity (Schneider and McGrew 2018). The location of Naming Speed tests in the current study was between Gwm and Gs subtests, suggesting that they measure a blend of attentional control and processing speed (Meyer and Reynolds 2018). It is also notable that these tests share letter and number content in addition to cognitive processes.

Last, with the Wechsler data, Oral Reading Fluency was in the reading cluster, but it was located near Digit Span and Letter-Number Sequencing auditory short-term storage tests within Gwm. Working memory predicts reading fluency in children with ADHD (Swanson and Siegel 2011) and SLD (Jacobson et al. 2011). The auditory short-term storage narrow ability within working memory is one component process that contributes to reading fluency and comprehension (Norton and Wolf 2012; Schneider and McGrew 2018). Oral Reading Fluency may have been closer to the auditory short-term storage tests than Word Reading and Pseudoword Decoding because the Oral Reading Fluency task contains context and connected text around each word that an examinee must hold in immediate awareness and manipulate to comprehend what has been read already and predict what is coming next. 

### 4.3. Regions and Fluency

McGrew et al. (2014) introduced a broader organization of tests, called regions, with MDS of the WJ IV: auditory-linguistic, figural-visual, reading-writing, quantitative-numeric regions, and speed-fluency. Auditory-linguistic, figural-visual, reading-writing, and quantitative-numeric regions were similarly visible in the WISC-V and WIAT-III map and each of the four Kaufman maps. These regions now have support from three different tests with three different samples. Some differences were found in the current study regarding speed-fluency, though these are likely due to test sampling differences. 

A speed-fluency region was visible in the WISC-V and WIAT-III MDS map. The WISC-V and WIAT-III speed-fluency region included three math fluency tests, two speed of lexical access tests, and three processing speed tests. The WISC-V and WIAT-III speed-fluency region did not include Oral Reading Fluency. The Oral Reading Fluency test measures speed and accuracy in reading. The reading skills and accuracy required in Oral Reading Fluency are also required in the other word reading tests and may explain why Oral Reading Fluency was in the reading-writing region instead of closer to the speed-fluency tests. It is notable that though the math fluency tests were near the other speed-fluency tests, the math fluency tests were in the overlapping space between the quantitative-numeric and speed-fluency regions.

Different from the WISC-V and WIAT-III map speed-fluency region, in each Kaufman map, a retrieval fluency region was evident. This region contained oral language tests of speed of lexical access or retrieval fluency that were separate from oral language tests in the auditory-linguistic area. The KABC-II does not include processing tests. The Kaufman reading fluency tests require examinees to read real and nonreal words in isolation instead of in sentences and were located in the reading-writing region, though they were on the side of the reading-writing region closest to the speed of lexical access tests. 

Locations of fluency tests in the MDS maps have implications for understanding academic difficulties related to fluency. Difficulties in reading and math fluency have been shown to co-occur, especially after second grade (Koponen et al. 2018) and it is necessary to understand whether disfluency come from a process deficit to intervene and remediate effectively. If fluency tests were located near each other in an academic fluency region of an MDS configuration of intelligence and academic achievement test scores, it would not lend causal evidence, but it would provide information about possible shared characteristics among fluency tests beyond academic content characteristics. In the WISC-V and WIAT-III MDS map and the WIAT-III only map, reading and math fluency tests were located near tests of the same academic domain. Based on results with these tests, fluency test scores should be kept with test of the same academic domain. Kaufman maps differed from WISC-V and WIAT-III regions in this study. There was not a speed-fluency region in the Kaufman maps even though Naming Facility and Associational Fluency tests from the KTEA-II are similar to the, the Naming Speed Literacy and Naming Speed Quantity tests from the WISC-V. The KABC-II does not include processing speed tests and KTEA-II reading fluency tests were closer to the reading-writing region. This meant that in the Kaufman maps, there was not a speed-fluency region. Instead, there was a narrower retrieval fluency region. The results support the split of Gr from the Glr cluster (Jewsbury and Bowden 2016). None of the MDS maps in this study included writing fluency tests, but analysis with additional academic fluency tests may result in different findings.

Additionally, academic fluency tests in the current study were not located between the higher-order thinking tests and basic skills tests as the conceptual framework from basic to higher-order academic skills would suggest (Mather and Wendling 2015). Basic skills tests and fluency tests are both simple tests in which examinees apply rules (they do not infer rules or relations between concepts). Fluency tests introduce speed to measure the automaticity with which the examinee completes the test. Though, fluency is not just speed of completing the task; accuracy matters too, so much that Paige and Magpuri-Lavell (2014) call it “accumaticity” in the context of reading fluency. Academic fluency tests were located around the periphery of the MDS map, suggesting that academic fluency tests in these assessments are simple, like the basic skill tests, and not of intermediate complexity between basic and higher-order tests. 

### 4.4. Content and Response Process Facets

Facets organize and define an observation. In the context of assessment, multiple facets define the tests that make up intelligence and academic achievement tests. Arithmetic is a numeric test (content facet), measuring latent fluid reasoning (cognitive operation facet), and examinees respond verbally (response process). Though complexity did not adequately describe test locations in the WISC-V and WIAT-III MDS configuration, follow-up analysis with these data revealed that additional features of the tests (different facets) described the placement of tests instead, consistent with previous MDS studies with intelligence tests alone (Cohen et al. 2006; Guttman and Levy 1991). 

In addition to interpreting CHC and academic clusters, the MDS maps appeared to systematically organize tests by shared content. Arithmetic’s highest correlations are with Math Problem Solving (.61), Math Fluency Subtraction (.59), Letter-Number Sequencing (.57), Digit Span (.50), and Numerical Operations (.46). These tests and the math fluency tests were in the center (and upper portion of the static view) in Figure 7, grouped by their numeric content. Complex reading, oral language, writing, and Gc tests were not in the center with Arithmetic and Math Problem Solving because they were grouped by verbal content (on the left side of the static view) in Figure 7. Tests with figural/pictorial content were on the other side of the tests with numeric content (lower right of the static view) in Figure 7. Tests were organized by the content facet (Cohen et al. 2006; Guttman and Levy 1991). 

MDS maps also appeared to organize tests in space by response mode processes. Tests with paper-pencil responses were grouped together (near the top of the static view) in Figure 8 and tests with verbal responses were grouped together (lower left of the static view) in Figure 8. Tests that allow verbal or manual response were near the one test (Block Design) that requires a manual response (lower right of the statice view) in Figure 8. When looking at test content and response process MDS maps together, it is notable that on the WISC-V and WIAT-III most of the verbal content tests also elicit verbal responses. Tests with other types of content (numeric; figural; letter, number, color, object) were mixed in requiring verbal, manual, or paper-pencil responses.

The AERA, APA, and NCME Standards for Educational and Psychological Testing (American Educational Research Association et al. 2014) include test content and response processes as sources of validity evidence in addition to internal structure, relations to other variables, and consequences of testing. This study supported content (Figure 7) and response process (Figure 8) validity. Previous MDS studies with Wechsler tests have demonstrated content and response facets in three dimensions (Guttman and Levy 1991) and two dimensions (Cohen et al. 2006); however, this is the first study to support content and response facets with the inclusion of academic achievement tests. Content and response process should be something that test users consider in interpretation of scores.

### 4.5. Limitations

This research is not without limitations. There are a few limitations regarding interpretation of the results. First, there is subjectivity in selecting the number of dimensions and in interpreting the resulting configurations. There is guidance about making an informed choice for the number of dimensions in terms of absolute fit; however, due to the limited research using MDS compared to other multivariate procedures, there is little precedent about appropriate model decisions such as number of dimensions. Further, more dimensions may exist, but it is difficult for humans to interpret findings beyond three dimensions (Ambinder et al. 2009). Visualizing and interpreting three-dimensional representations is difficult because visual information is first recorded on the retina in two dimensions and then depth is integrated (Finlayson et al. 2017). Additionally, there are capacity limits to visual working memory (Qian and Zhang 2019). There is also subjectivity in the interpretations, but the visual exploration of results also creates opportunities to examine additional facets or dimensions, like the response processes in Figure 8.

Another limitation is related to the analysis of complexity. Calculating the center of the configurations via the mean is sensitive to outliers (like the Cancellation test that is far from all other tests). The center point that represents the mean of each dimension does not necessarily represent the center of the densest part of the configuration. 

Next, there were limitations related to the generalization of the results. United States demographics have changed since data were collected in the norming procedures for the measures, so the findings may not generalize to the current population. Additionally, the sample represents the English-speaking U.S. population, so the findings cannot be generalized to those whose first language is not English (see Ortiz et al. 2018). 

### 4.6. Future Research

In the future, it would be informative to include multiple intelligence or academic achievement test batteries or narrower measures. If CHC ability clusters and academic content clusters could be more balanced (e.g., each CHC ability and academic area is measured with each response mode), correlations sampled more ability areas (i.e., more CHC narrow abilities), or included more variety within clusters (e.g., four ways to measure Gv) cross-battery comparisons would be more accurate and implications could extend beyond the boundaries of a given test battery (Beauducel and Kersting 2002; Süß and Beauducel 2015). More balance among measures could also help to eliminate alternative explanations for some findings in this research, such as having a disproportionate number of reading tests in the analysis, which may affect the findings. 

MDS will also be useful as an exploratory technique when abilities are considered for addition to the CHC taxonomy (such as Gei or Emotional Intelligence per Schneider and McGrew 2018) or to test new theoretical frameworks, such as process overlap theory to explain *g* (Conway et al. 2021). Given its unrestricted nature, it may help some with overly restricted thinking.

There is also a need to replicate with non-standardization samples (Graves et al. 2020). MDS could be useful with data from culturally and linguistically diverse students. Verbal content and response processes that may increase complexity or change task demands depending on an individual’s language skills. For example, the working memory task demands of a reading comprehension test may be higher if an individual is focusing on word- or sentence-level understanding (Acosta Caballero 2012). Given that the regions (e.g., auditory-linguistic) have emerged across multiple tests across multiple samples, it would be interesting to investigate if these findings are invariant across non-representative samples.

CHC theory is a flexible taxonomy and analysis of constructs that are being considered for inclusion is another direction for future research. Broader regions, like McGrew et al. (2014) auditory-linguistic, reading-writing, quantitative-numeric, and speed-fluency regions that were supported in the current study should also be studied more with additional assessments. Emotional intelligence and sensory domains (tactile, kinesthetic, etc.) are not typically included in comprehensive psychoeducational evaluations. These and other areas of abilities should be analyzed in future studies for a more complete model of individual differences that are relevant in school, occupations, and creative pursuits. 

Finally, it would be worthwhile to apply MDS analysis of variables representing individual differences across a larger time span than a few weeks between test administrations. It would be interesting to also develop a method for representing longitudinal variables in continuous geometric space.

### 4.7. Implications

The results from this research suggest theoretical and practical implications. Practitioners need to be aware of shared content and response processes across tests that convey similarities beyond cognitive processes. Test content and response processes are discussed in test manuals, but it is not clear how often they are considered in practice. One interpretive practice that is not supported by this research is a fluency score that cuts across academic areas. More information is needed about how fluency relates to memory processes, basic skills acquisition, and higher-order academic achievement skills (Gerst et al. 2021; Lovett et al. 2020). 

## 5. Conclusions

Snow et al. (1984, p. 47) called MDS maps of intelligence and academic achievement correlations “The Topography of Ability and Learning Correlations.” MDS configurations give a sense of the landscape that is at times obscured by the viewfinder of other multivariate analyses, like factor analysis. 

Several important findings emerged from this MDS research. First, test organization by CHC ability factors and academic achievement domains was supported, and in that respect, findings were consistent with findings from factor analysis (Carroll 1993). CHC theory was a useful way to describe the findings. Second, in addition to organization by CHC ability factors and academic domains, broader regions were visible, supporting McGrew et al. (2014) findings. Third, content and response process facets are useful in understanding intelligence tests and achievement test score correlations. Practitioners need to be aware of how test information is presented to examinees and how their responses are elicited. Last, academic fluency tests were not as distinctly or consistently located in a speed-fluency region in the test batteries examined in this study because they were near academic domains.

## Figures and Tables

**Table 1:** Demographic Information: WISC-V and WIAT-III Validity Sample, WISC-V Norming Sample.

Demographic Variable	% of Validity Sample *N* = 181
Sex	
Female	44.8
Male	55.2
Race/Ethnicity	
Asian	1.7
Black	19.9
Hispanic	21.0
Other	7.2
White	50.3
Highest Parental Education	
Grade 8 or less	2.2
Grade 9–12, no diploma	8.3
Graduated high school or GED	24.9
Some College/Associate Degree	35.4
Undergraduate, Graduate, or Professional degree	29.3

**Table 2:** Demographic Information: Kaufman (KABC-II and KTEA-II) Subsamples, Full Sample.

Kaufman Test Demographic Information: KABC-II and KTEA-II Grade Subsamples				
	Grades 1–3	Grades 4–6	Grades 7–9	Grades 10–12
Sex	(*n* = 592)	(*n* = 558)	(*n* = 566)	(*n* = 401)
Female	49.3	48.9	49.5	50.9
Male	50.7	51.1	50.5	49.1
Ethnicity				
Black	15.5	13.8	15.5	13.7
Hispanic	19.9	18.3	15.4	17.2
Other	4.7	6.1	5.7	5.5
White	59.8	61.8	63.4	63.6
Highest Parent Ed.				
Grade 11 or less	13.0	16.5	14.8	15.5
HS graduate	32.6	31.9	32.2	33.4
1–3 years college	31.9	28.7	29.3	28.4
4 year degree+	22.5	22.9	23.7	22.7
Geographic Region				
Northeast	16.6	16.5	11.3	9.5
North central	23.6	27.1	23.0	27.9
South	35.5	33.2	35.0	35.9
West	24.3	23.3	30.7	26.7
Age Band				
6:00–6:11	20.6			
7:00–7:11	30.1			
8:00–8:11	31.9	0.2		
9:00–9:11	16.7	16.8		
10:00–10:11	0.7	33.7		
11:00–11:11		33.3	0.2	
12:00–12:11		14.7	20.5	
13:00–13:11		1.1	32.3	
14:00–14:11		0.2	32.0	0.5
15:00–15:11			13.4	15.5
16:00–16:11			0.9	32.9
17:00–17:11			0.4	33.4
18:00–18:11			0.2	17.5
19:00–19:11			0.2	0.2

**Table 3:** Ordinal and Interval MDS Stress Comparisons in Two and Three Dimensions.

Correlation Matrix	Ordinal, Two Dimensions	Interval, Two Dimensions	Ordinal, Three Dimensions	Interval, Three Dimensions
WISC-V and WIAT-III	0.22	0.26	**0.15**	0.18
Kaufman Grades 1–3	0.24	0.29	**0.14**	0.20
Kaufman Grades 4–6	0.24	0.29	**0.14**	0.20
Kaufman Grades 7–9	0.18	0.20	**0.11**	0.18
Kaufman Grades 10–12	0.18	0.20	**0.13**	0.18

Note. MDS models with “Torgerson” classical scaling starting configuration. Bolded numbers were the lowest stress values among the four configuration for that matrix.

**Table 4:** WIAT-III and WISC-V Subtests Ordered by Distance from Center of 3D Configuration.

Subtest Abbr.	Subtest	Composite	*g*-Loading or Complexity	Distance from Center
AR	Arithmetic	Fluid Reasoning	.73	0.09
MPS	Math Problem Solving	Mathematics	High	0.24
LN	Letter-Number Sequencing	Working Memory	.65	0.26
NO	Numerical Operations	Mathematics	Low	0.39
SP	Spelling	Written Expression	Low	0.40
DS	Digit Span	Working Memory	.66	0.40
OE	Oral Expression	Oral Language	High	0.43
SI	Similarities	Verbal Comprehension	.72	0.45
LC	Listening Comprehension	Oral Language	High	0.50
RC	Reading Comprehension	Reading Comp. & Fluency	High	0.50
MFS	Math Fluency Subtraction	Math Fluency	Medium	0.53
WR	Word Reading	Basic Reading	Low	0.56
SC	Sentence Composition	Written Expression	High	0.56
VC	Vocabulary	Verbal Comprehension	.73	0.62
IN	Information	Verbal Comprehension	.72	0.63
CO	Comprehension	Verbal Comprehension	.63	0.63
DST	Delayed Symbol Translation	Symbol Translation		0.66
PD	Pseudoword Decoding	Basic Reading	Low	0.67
ORF	Oral Reading Fluency	Reading Comp. & Fluency	Medium	0.68
RST	Recognition Symbol Translation	Symbol Translation		0.68
PS	Picture Span	Working Memory	.55	0.70
IST	Immediate Symbol Translation	Symbol Translation		0.74
FW	Figure Weights	Fluid Reasoning	.64	0.78
NSQ	Naming Speed Quantity	Naming Speed		0.78
BD	Block Design	Visual Spatial	.64	0.80
MFA	Math Fluency Addition	Math Fluency	Medium	0.80
CD	Coding	Processing Speed	.37	0.81
VP	Visual Puzzles	Visual Spatial	.66	0.82
MR	Matrix Reasoning	Fluid Reasoning	.64	0.82
PC	Picture Concepts	Fluid Reasoning	.54	0.82
SS	Symbol Search	Processing Speed	.42	0.82
MFM	Math Fluency Multiplication	Math Fluency	Medium	0.85
EC	Essay Composition	Written Expression	High	0.86
NSL	Naming Speed Literacy	Naming Speed		1.06
CA	Cancellation	Processing Speed	.19	1.33

**Table 5:** Kaufman Subtests Ordered by Distance from Center of 3D Configuration (Grades 4–6).

Subtest Abbr.	Subtest	Composite	*g*-Loading or Complexity	Distance from Center
RC	Reading Comprehension	Reading	High	0.05
MA	Math Concepts & Applications	Mathematics	High	0.18
WE	Written Expression	Written Language	High	0.21
WR	Letter & Word Recognition	Reading, Decoding	Low	0.24
TW	Word Recognition Fluency	Reading Fluency	Medium	0.27
RI	Riddles	Gc	0.72	0.28
VK	Verbal Knowledge	Gc	0.71	0.37
SP	Spelling	Written Language	Low	0.41
PR	Pattern Reasoning	Gf	0.7	0.43
DE	Nonsense Word Decoding	Sound-Symbol, Decoding	Low	0.45
MC	Math Computation	Mathematics	Low	0.46
EV	Expressive Vocabulary	Gc	0.67	0.47
OE	Oral Expression	Oral Language	High	0.47
TD	Decoding Fluency	Reading Fluency	Medium	0.53
RL	Rebus	Glr	0.67	0.56
LC	Listening Comprehension	Oral Language	High	0.61
RD	Rebus Delayed	Glr	0.64	0.63
TR	Triangles	Gv	0.6	0.64
PS	Phonological Awareness	Sound-Symbol	Low	0.66
PL	Phonological Awareness (Long)	Sound-Symbol	Low	0.69
AF	Associational Fluency	Oral Fluency	Low	0.77
HM	Hand Movements	Gsm	0.52	0.79
AT	Atlantis	Glr	0.54	0.8
WO	Word Order	Gsm	0.54	0.8
NR	Number Recall	Gsm	0.44	0.86
RO	Rover	Gv	0.53	0.89
ST	Story Completion	Gf	0.61	1.03
BC	Block Counting	Gv	0.54	1.06
AD	Atlantis Delayed	Glr	0.43	1.12
R2	Naming Facility: Objects, Colors, & Letters	Oral Fluency	Low	1.21
R1	Naming Facility: Objects & Colors	Oral Fluency	Low	1.22

## Data Availability

The data presented in this study are not publicly available because they are confidential and proprietary.

## Supplementary Materials

The following supporting information can be downloaded at: https://www.mdpi.com/article/10.3390/jintelligence10040117/s1. File S1: Multidimensional Scaling of Cognitive Ability and Academic Achievement: Supplemental Figures.
