# Supplementary material for: Multidimensional Scaling of Cognitive Ability and Academic Achievement Scores
Source: J Intell. 2022 Dec 1;10(4):117. doi: 10.3390/jintelligence10040117 (PMC9785841; doi:10.3390/jintelligence10040117)
Supplement: Supplementary file 1 [file jintelligence-10-00117-s001.zip › jintelligence-1972189-supplementary.html]

Multidimensional Scaling with ACH and COG Scores: Supplemental Figures


# Multidimensional Scaling of Cognitive Ability and Academic Achievement: Supplemental Figures

#### 9/18/2022

##### Standardization data and analysis from the Kaufman Test of Educational Achievement, Second Edition-Comprehensive Form (KTEA-2). Copyright © 2004 NCS Pearson, Inc. Used with permission. All rights reserved.

##### Standardization data and analysis from the Kaufman Assessment Battery for Children, Second Edition (KABC-II). Copyright © 2004 NCS Pearson, Inc. Used with permission. All rights reserved.

##### Standardization data from the Wechsler Intelligence Scale for Children, Fifth Edition (WISC-5). Copyright © 2014 NCS Pearson, Inc. Used with permission. All rights reserved.


### Figure 1 WISC-V and WIAT-III 3D MDS Configuration, Color-Coded by Complexity

|  |  |  |  |  |  |
| --- | --- | --- | --- | --- | --- |
| \_\_ | Basic Skills - Low Complexity | \_\_ | Fluency Skills - Med Complexity | \_\_ | Higher-Order - High Complexity |

|  |  |  |  |  |  |
| --- | --- | --- | --- | --- | --- |
| \_\_ | g-loading less than .60 | \_\_ | g-loading ≥.60 | \_\_ | g-loading ≥.70 |


### Figure 2 WISC-V and WIAT-III 3D MDS Configuration CHC and Academic Clusters

|  |  |  |  |  |  |  |  |  |  |  |  |  |  |
| --- | --- | --- | --- | --- | --- | --- | --- | --- | --- | --- | --- | --- | --- |
| \_\_ | Gc, VCI | \_\_ | Gv, VSI | \_\_ | Gf, FRI | \_\_ | Gwm, WMI | \_\_ | Gs, PSI | \_\_ | NSI | \_\_ | STI |
| \_\_ | Oral Lang | \_\_ | Reading | \_\_ | Writing | \_\_ | Math |


### Figure 4 Kaufman Grades 4-6 3D MDS Configuration, Color-Coded by Complexity

|  |  |  |  |  |  |
| --- | --- | --- | --- | --- | --- |
| \_\_ | Basic Skills - Low Complexity | \_\_ | Fluency Skills - Med Complexity | \_\_ | Higher-Order - High Complexity |

|  |  |  |  |  |  |
| --- | --- | --- | --- | --- | --- |
| \_\_ | g-loading less than .60 | \_\_ | g-loading ≥.60 | \_\_ | g-loading ≥.70 |


### Figure 5 Kaufman Grades 4-6 3D MDS Configuration CHC and Academic Clusters

|  |  |  |  |  |  |  |  |  |  |
| --- | --- | --- | --- | --- | --- | --- | --- | --- | --- |
| \_\_ | Gc | \_\_ | Gv | \_\_ | Gf | \_\_ | Gsm | \_\_ | Glr |
| \_\_ | Oral Lang | \_\_ | Reading | \_\_ | Writing | \_\_ | Math |

### Figure 7 WISC-V and WIAT-III 3D MDS Configuration, Color-Coded by Content

|  |  |  |  |  |  |  |  |
| --- | --- | --- | --- | --- | --- | --- | --- |
| \_\_ | Verbal content | \_\_ | Numeric content | \_\_ | Letter, Number, Color, Object content | \_\_ | Figural content |


### Figure 8 WISC-V and WIAT-III 3D MDS Configuration, Color-Coded by Response Mode

|  |  |  |  |  |  |  |  |
| --- | --- | --- | --- | --- | --- | --- | --- |
| \_\_ | Verbal response | \_\_ | Manual or verbal response | \_\_ | Manual response | \_\_ | Paper-pencil response |


### Kaufman Grades 1-3 3D MDS Configuration, Color-Coded by Complexity

|  |  |  |  |  |  |
| --- | --- | --- | --- | --- | --- |
| \_\_ | Basic Skills - Low Complexity | \_\_ | Fluency Skills - Med Complexity | \_\_ | Higher-Order - High Complexity |

|  |  |  |  |  |  |
| --- | --- | --- | --- | --- | --- |
| \_\_ | g-loading less than .60 | \_\_ | g-loading ≥.60 | \_\_ | g-loading ≥.70 |


### Kaufman Grades 1-3 3D MDS Configuration CHC and Academic Clusters

|  |  |  |  |  |  |  |  |  |  |
| --- | --- | --- | --- | --- | --- | --- | --- | --- | --- |
| \_\_ | Gc | \_\_ | Gv | \_\_ | Gf | \_\_ | Gsm | \_\_ | Glr |
| \_\_ | Oral Lang | \_\_ | Reading | \_\_ | Writing | \_\_ | Math |


### Kaufman Grades 7-9 3D MDS Configuration, Color-Coded by Complexity

|  |  |  |  |  |  |
| --- | --- | --- | --- | --- | --- |
| \_\_ | Basic Skills - Low Complexity | \_\_ | Fluency Skills - Med Complexity | \_\_ | Higher-Order - High Complexity |

|  |  |  |  |  |  |
| --- | --- | --- | --- | --- | --- |
| \_\_ | g-loading less than .60 | \_\_ | g-loading ≥.60 | \_\_ | g-loading ≥.70 |


### Kaufman Grades 7-9 3D MDS Configuration CHC and Academic Clusters

|  |  |  |  |  |  |  |  |  |  |
| --- | --- | --- | --- | --- | --- | --- | --- | --- | --- |
| \_\_ | Gc | \_\_ | Gv | \_\_ | Gf | \_\_ | Gsm | \_\_ | Glr |
| \_\_ | Oral Lang | \_\_ | Reading | \_\_ | Writing | \_\_ | Math |


### Kaufman Grades 10-12 3D MDS Configuration, Color-Coded by Complexity

|  |  |  |  |  |  |
| --- | --- | --- | --- | --- | --- |
| \_\_ | Basic Skills - Low Complexity | \_\_ | Fluency Skills - Med Complexity | \_\_ | Higher-Order - High Complexity |

|  |  |  |  |  |  |
| --- | --- | --- | --- | --- | --- |
| \_\_ | g-loading less than .60 | \_\_ | g-loading ≥.60 | \_\_ | g-loading ≥.70 |


### Kaufman Grades 10-12 3D MDS Configuration CHC and Academic Clusters

|  |  |  |  |  |  |  |  |  |  |
| --- | --- | --- | --- | --- | --- | --- | --- | --- | --- |
| \_\_ | Gc | \_\_ | Gv | \_\_ | Gf | \_\_ | Gsm | \_\_ | Glr |
| \_\_ | Oral Lang | \_\_ | Reading | \_\_ | Writing | \_\_ | Math |
